# Exocrine Pancreatic Insufficiency in Type 1 and Type 2 Diabetes

**DOI:** 10.1007/s11892-020-01304-0

**Published:** 2020-04-01

**Authors:** Bernhard Radlinger, Gabriele Ramoser, Susanne Kaser

**Affiliations:** 1 Department of Internal Medicine 1, Medical University Innsbruck, Anichstrasse 35, 6020 Innsbruck, Austria; 2 Department of Pediatrics II, Medical University Innsbruck, Innsbruck, Austria

**Keywords:** Diabetes mellitus, Exocrine pancreatic insufficiency, Fecal elastase, Islet-acinar axis

## Abstract

**Purpose of Review:**

Type 1 and type 2 diabetes are often accompanied by mostly mild forms of exocrine pancreatic insufficiency. Despite high prevalence, little is known about the clinical consequences of exocrine pancreatic insufficiency and its optimal (nutritional) treatment. Even less is known if and to what extent exocrine pancreas insufficiency also affects glycemic control in diabetes. This article aims for summarizing current clinical knowledge on screening, diagnosis, and treatment and gives an overview on the pathophysiology of exocrine pancreatic insufficiency in diabetes.

**Recent Findings:**

Recent studies reveal novel insights into the close interaction of acinar, ductal, and endocrine cells and the gut-pancreas axis.

**Summary:**

Exocrine pancreatic insufficiency is a clinically relevant, frequent but poorly understood disorder in both type 1 and type 2 diabetes.

## Introduction

Exocrine pancreatic insufficiency (EPI or pancreatic exocrine insufficiency (PEI)) also named exocrine pancreatic dysfunction (EPD) or pancreatic maldigestion is defined as malabsorption resulting from insufficient digestion of nutrients, especially fats [1, 2]. EPI is caused by insufficient secretion of pancreatic enzymes such as amylase, lipase, and protease and/or sodium bicarbonate. Clinical symptoms including steatorrhea, weight loss, excess flatulence, abdominal discomfort and clinical signs of vitamin (A, D, E, K), and albumin deficiency usually occur when pancreatic enzyme activity is lower than 10% [2–4].

## Prevalence

While the frequency of EPI in otherwise healthy persons is unclear [3], several disorders including different types of diabetes are associated with significantly increased risk for EPI. By definition, EPI is found in nearly all patients with pancreatogenic diabetes [5], therefore studies on the prevalence of EPI in patients with diabetes are typically focused on type 1 and type 2 diabetes. In the past, EPI diagnosed via direct pancreatic function tests was thought to be present in about 50% of patients with diabetes [6]. However, direct pancreatic function tests were replaced by non-invasive, cheaper tests showing partly different EPI frequency rates [6]. Nowadays, determination of fecal elastase-1 (FE-1) concentration is most frequently used for diagnosing EPI in clinical and epidemiological studies. FE-1 is regarded as a suitable indicator of pancreatic enzyme secretion as it passes the intestinal tract in an unmodified way and does not get secreted/ingested past the pancreas [7].

In a recent study, 12.7% of patients with diabetes were diagnosed with EPI as defined by FE-1 levels < 200 μg/g stool. Prevalence of EPI was higher in type 1 diabetes than in type 2 diabetes [8]. In this study, diabetes duration turned out to be a risk factor of EPI, while in others, no association between diabetes duration and EPI was found [9–11]. In contrast to this small sample size study, Hardt and colleagues [12] reported reduced FE-1 concentrations in 40.7% of patients with long-standing diabetes. Frequency rates were comparable between patients with either type 1 or type 2 diabetes, and type of treatment had no effect on EPI risk in patients with type 2 diabetes [12]. Lower EPI rates were reported by Larger and colleagues [13] who found that in total 20.3% of patients suffering from either type 1 or type 2 diabetes had significantly reduced FE-1 levels. No information on severity of symptoms in affected patients was available from these studies.

In a meta-analysis of 17 studies including 3662 subjects with diabetes, EPI—defined by decreased FE-1 concentrations ≤ 200 μg/g stool—was reported in 38.62% of patients with type 1 diabetes and 28.12% of patients with type 2 diabetes [14]. Remarkably, in a previous study of patients with type 1 diabetes, 40% had a normal fat excretion (< 7 g/day on a 100 g fat/day diet) despite markedly decreased FE-1 levels (≤ 100 μg/g stool). On the other hand, only 12% of patients with significantly reduced FE-1 levels had strongly increased fecal fat excretion of more than 15 g/day [15]. Similarly, in another study, only 8 from 19 patients with type 1 diabetes with fecal fat excretions > 7 g/day had FE-1 levels ≤ 200 μg/ g stool, while fecal fat excretions > 7 g/day were found in only 5 of 7 patients with FE-1 concentrations ≤ 100 μg/g stool [9]. In accordance with these findings, Hahn and colleagues suggested that neither FE-1 concentrations nor increased fecal fat levels were reliable predictors of EPI as diagnosed by the gold standard secretin-cerulein test in patients with type 1 diabetes [16].

In type 2 diabetes, neither indigestion nor diarrhea is more frequent in patients with low FE-1 concentrations when compared with those with intermediate or normal FE-1 levels. However, 25-OH-vitamin D and lipase concentrations were significantly decreased in patients with low FE-1 concentrations [17].

Bariatric surgery is considered as additional and optional treatment in obese patients with type 2 diabetes. Notably, in a cohort of mainly non-diabetic obese subjects, 31% of patients developed EPI after Roux-en-Y gastric bypass surgery [18].

## Clinical Consequences of EPI

**Fig. 1:**
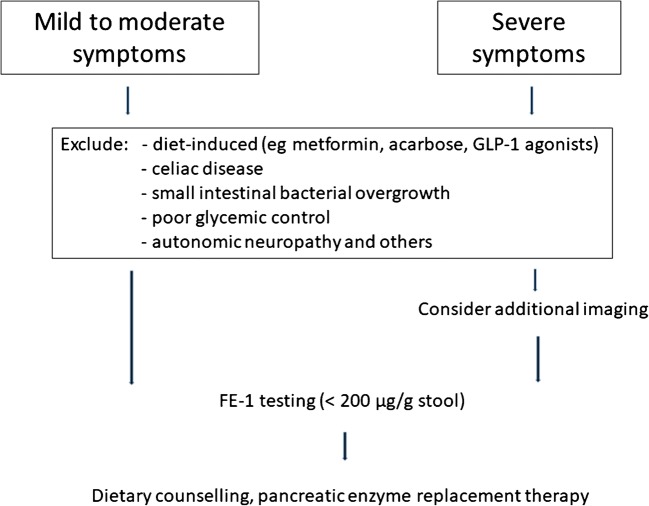
Proposed diagnostic approach in symptomatic patients with diabetes. Adapted from Kaser S et al. [75]

Symptoms of EPI are usually mild to moderate in affected patients with diabetes. In severe disease, these include steatorrhea and weight loss, while in mild and moderate disease, typically abdominal discomfort or pain, diarrhea, and flatulence might be misdiagnosed as drug-induced (metformin, glucagon-like peptide 1 (GLP-1) agonists) (Fig. 1) [6]. Studies showing only weak correlations between fecal fat excretion, functional pancreas tests and FE-1 levels in patients with diabetes further underline the necessity of considering other differential diagnosis of steatorrhea such as celiac disease, bacterial overgrowth in the proximal small bowel, or poor glycemic control in patients with diabetes [9, 16]. In a very recent population-based study, a close association between pancreatic elastase and microbial diversity was shown underlining the complex interaction between gut and exocrine pancreas. In this study patients with EPI defined as FE-1 < 200 μg/g stool showed an overall less diverse microbial composition and an increase in species associated with chronic inflammation. Results were also correlated with factors like age, sex, smoking, BMI, and dietary habits, however, none of these factors explained more changes in the bacterial composition than did fecal elastase levels [19••].

In patients with EPI due to chronic pancreatitis, increased rates of deficiencies of vitamins E, D, and K as well as osteopenia and osteoporosis were reported [20]. In another study, presence of EPI was associated with sarcopenia [21]. However, from the literature, it is not clear whether these data are applicable to diabetic patients with EPI also.

## Pathophysiological Aspects of EPI in Diabetes

The etiology of EPI in diabetes is not fully clear yet. In the literature, pathophysiological aspects were more often investigated in settings of (autoimmune) insulin deprivation than in states of insulin resistance and hyperinsulinemia. Proposed factors include diminished trophic effects of insulin, inflammation, fibrosis, and steatosis which are summarized in detail here. Additionally, diabetic microangiopathy leading to ischemia in the exocrine pancreas and impaired enteropancreatic reflexes due to autonomic neuropathy have been discussed controversially to contribute to the development of EPI [6, 22–24].

### Islet-Acinar Axis

**Fig. 2:**
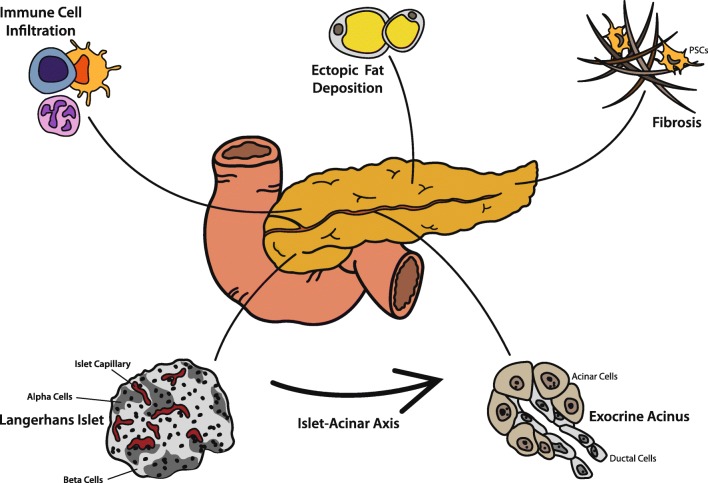
Contributing factors to exocrine pancreatic insufficiency in patients with diabetes. Due to portal vessels supplying exocrine acini with the *secretome* of endocrine islets, acinar cells are under endocrine control, in the short-term for well-regulated upregulation of pancreatic enzyme secretion and in the long-term for trophic control of both acinar cells and ductal cells. Overall and with time, the pancreata in patients with type 1 or type 2 diabetes get atrophic and exocrine function declines. Islet microvasculature might be affected by diabetic microangiopathy, and diabetic neuropathy is also discussed to influence this islet-acinar axis (not depicted specifically in this figure). Immune cell infiltration (mainly CD8+, CD4+, and CD11c+ cells) and autoantibodies targeting the exocrine compartment are frequently found in both type 1 and in type 2 diabetes. A rise in collagen deposition and loss of extracellular matrix remodeling facilitated by activated pancreatic stellate cells (PSCs) lead to an increased rate of pancreatic fibrosis in patients with diabetes. Additionally, ectopic fat accumulation has been discussed to contribute to development of EPI, however, data from the literature are controversial

Already in 1962, Hellman and colleagues reported a *halo* effect in detail around pancreatic islets [25] which results from islet surrounding acinar cells being the first cells to get in contact with the islet’s *secretome* via a capillary network. The intricate vascular anatomy of the pancreas comprises of islets receiving an over proportional amount of arterial blood compared with the exocrine compartment and the exocrine pancreas receiving a fair share of this blood directly via special islet-acinar portal vessels [26–29] (Fig. 2). Due to the nature of the vascular system in the pancreas, local *peri-islet* insulin levels are likely to be very high compared with systemic levels, considering that even after dilution into the portal vein, insulin levels are roughly twice as high as compared with systemic circulation [30]. The trophic effect of insulin on acinar cells is well established: insulin downregulates its specific receptor in acinar cells and upregulates the synthesis of digestive enzymes [31–33]. Additionally, glucose uptake is increased upon insulin exposure to acinar cells [34]. Underlining the trophic effects of insulin on acinar cells, acinar atrophy was most pronounced around insulin-deficient islets compared with insulin expressing islets in autopsies of patients with type 1 diabetes [35].

Interestingly, type 2 diabetes and obesity are associated with increased pancreatic duct cell replication [36] and not until recently it has been shown that in states of increased insulin demand, ductal cells contribute to the compensatory β cell pool by differentiation and/or neogenesis [37••].

Besides insulin, endocrine pancreatic islets also produce glucagon, somatostatin, ghrelin, pancreatic polypeptide, and other peptide hormones, which are involved in the regulation of the islet-acinar axis. Dysregulation or deficiency of these hormones in diabetes is thought to also contribute to EPI. Detailed biological effects of these substances on nearby acinar cells have been reviewed in detail recently [38].

Despite the delicate intra-organ crosstalk between islets and exocrine acini, there is a considerable size difference between these two compartments with islets only making 1–2% of total pancreas mass [39]. Therefore, size estimates of total pancreas mass, either with direct measurement from autopsies, ultrasound scans, or with CT/MRI scans, mainly reflect the size of the exocrine pancreas. Remarkably, total, parenchymal, and fat volume increases with obesity, while no enlargement of fat volume were reported in patients with type 2 diabetes [40].

In general, the mean pancreas size of patients with type 1 diabetes is significantly decreased [41], probably resulting from lacking trophic effects of insulin. However, also the pancreata of patients with type 2 diabetes are smaller when compared with healthy controls [42]. Interestingly, Philippe et al. reported that reduced pancreas size in patients with either type 1 or type 2 diabetes is also associated with presence of exocrine pancreatic insufficiency [43].

### Fibrosis and Steatosis

Besides a mere change in size, studies in different diabetes animal models suggest a loss of extracellular matrix remodeling with a focus in collagen deposition, especially at the islet-acinar interface, together with increased angiogenesis [26, 44, 45]. Accordingly, a recent meta-analysis reported that 59.4% of patients with either type 1 or type 2 diabetes show histopathological signs of fibrosis in the exocrine part of the pancreas [14]. Mechanistically, pancreatic stellate cells (PSC) seem to be crucial drivers of pancreatic fibrosis. Cytokines such as platelet-derived growth factor (PDGF) and transforming growth factor β (TGF-β) and hyperglycemia lead to activation of PSC resulting in increased collagen production [46–48]. Besides fibrosis, diabetes is also associated with ectopic fat deposition in the pancreas. From the literature, it is unclear whether pancreatic steatosis is a cause or consequence of β cell failure, hyperglycemia, and increased levels of fatty acids [49, 50]. Its role in exocrine pancreatic insufficiency is even less clear: association studies between pancreatic fat content and FE-1 levels showed mixed results [51, 52].

### Inflammation

While immune cell infiltration of islets in type 1 diabetes is well known, Rodriguez-Calvo et al. [53] found high CD8+ T cells infiltration in the exocrine pancreas of type 1 diabetic patients also. Remarkably, high immune cell infiltration of the exocrine compartment was reported even without apparent insulitis in patients with type 1 diabetes. In long-standing diabetes, not only CD8+ T cells but also CD4+ T cells and CD11c+ cells were found in the exocrine pancreas. The pathophysiological role of immune cells in acinar atrophy and fibrosis is not clear. Noteworthy, type 2 diabetes is also associated with increased immune cell infiltration of the exocrine pancreas [53, 54].

Additionally, autoantibodies raised against exocrine antigens have been observed in up to 39% of patients with type 1 diabetes and in 0.9% of patients with type 2 diabetes [54]. Autoantibodies targeting the pancreatic enzyme bile salt–dependent lipase were found in 73.5% of patients with type 1 diabetes [55].

## Diagnostic Approach and Treatment

Invasive, expensive and time-consuming functional pancreatic tests including the secretin-cholecystokinin (CCK) or secretin-cerulein stimulation tests were considered as gold standard for diagnosis of EPI in the past [56]. Nowadays, the 72-h fecal fat test serves as gold standard for the quantification of steatorrhea, however, it has significant limitations in detecting mild to moderate EPI [7]. ^13^C-breath tests including the ^13^C-mixed triglyceride breath test are also time-consuming and thus not frequently used in daily clinical routine [57–60].

In clinical practice, determination of FE-1 is commonly used as a marker of EPI. Elastase-1 is synthesized and secreted by pancreatic acinar cells and then passes the intestinal tract with minimal degradation after binding to bile salts [7]. Fecal levels of elastase-1 highly correlate with pancreatic elastase-1 secretion but also with amylase, lipase, and trypsin [61, 62]. False positive results are common in patients with diarrhea and might be avoided by lyophilization of feces and adjustment to a standardized water content [63]. Sensitivity and specificity of the FE-1 test is highly dependent on the severity of symptoms. A FE-1 cutoff ≤ 200 μg/g stool indicates EPI, while FE-1 concentrations of less than ≤ 100 μg/g stool are indicative of severe EPI [64]. In subjects with pancreatic disease, the mean sensitivity using a cutoff ≤ 200 μg/g stool was 63% in mild deficiency and 100% in moderate-to-severe EPI with a specificity of 93% when compared with the secretin-CCK or secretin-cerulein test [65]. In a meta-analysis of 8 studies, the sensitivity of the FE-1 test was calculated as 54% for mild, 75% for moderate, and 95% for severe EPI, while overall specificity was 79% in this study [66]. Considering the high prevalence of EPI in patients with diabetes, screening of symptomatic patients, especially those with nutritional deficiencies using the FE-1 test might be useful [67].

Therapy of EPI is based on both dietary counseling and administration of exogenous pancreatic enzymes. Generally, fat restriction and very high-fiber diets are recommended to be avoided in symptomatic EPI [67, 68]. Substitution of fat-soluble vitamins and other micronutrients (especially vitamin B12) might be necessary [67, 68]. Individual dosing of pancreatic enzyme replacement with at least 40,000 U of lipase per main meal and 10,000–25,000 U/snack is recommended for treatment of patients with EPI [69]. Notably, no specific dietary recommendations for patients with diabetes and EPI are available from interventional studies. In two previous studies, replacement therapy was associated with improved glycemic control or increased insulin and incretin response in patients with pancreatic disease and glucose intolerance [70, 71]. However, in a prospective study in patients with type 1 diabetes, enzyme replacement did not improve glycemic control [72].

## Conclusions

EPI is highly prevalent in type 1 diabetes and common in type 2 diabetes, however, symptoms are usually mild and not necessarily reflected by increased fat excretion and reduced FE-1 levels underlining the necessity of improved diagnostic tests or algorithms. In patients with pancreatic disease, EPI often goes hand in hand in weight loss, malnutrition, osteoporosis, sarcopenia, and even increased mortality [73, 74]. The clinical consequences and optimal dietary treatment in patients with diabetes are less clear and need further investigations. The pathophysiology of EPI in type 1 and type 2 diabetes is not fully clear but includes inflammation, fibrosis, and steatosis of the exocrine pancreas and disturbances in the islet-acinar axis especially decreased trophic effects of insulin. In conclusion, although highly prevalent, EPI in both type 1 and type 2 diabetes remains a poorly understood disease of unclear clinical long-term significance.
